# Correlations of health literacy with individuals’ understanding and use of medications in Southern Taiwan

**DOI:** 10.1515/med-2025-1203

**Published:** 2025-07-08

**Authors:** Pin-Tzu Chen, Min-Li Chen, Li-Chun Chang, Chin-Ho Kuo, Yu-Hsiang Kuan

**Affiliations:** Division of Hematology-Oncology, Ditmanson Medical Foundation Chia-Yi Christian Hospital, Chiayi, 60002, Taiwan; Graduate Institute of Nursing, Chang Gung University of Science and Technology, Chiayi County, Taiwan; Department of Nursing, Chang Gung University of Science and Technology, Taoyuan, Taiwan; Department of Pharmacology, School of Medicine, Chung Shan Medical University, No. 110, Sector 1, Jianguo N. Road, Taichung, 11662, Taiwan; Department of Pharmacy, Chung Shan Medical University Hospital, Taichung, 11662, Taiwan; Division of Hematology-Oncology, Ditmanson Medical Foundation Chia-Yi Christian Hospital, No. 539, Zhongxiao Road, Chiayi, 60002, Taiwan

**Keywords:** health literacy, medication adherence, treatment adherence and compliance, Southern Taiwan, treatment outcome, health status, cross-sectional studies

## Abstract

**Background:**

Investigated the relationship between health literacy and treatment adherence and compliance due to incorrect medication use resulting from low health literacy in southern Taiwan.

**Methods:**

A cross-sectional study in Chiayi, Taiwan (Sep–Nov 2013) used randomized sampling to survey 300 adults. Health literacy was assessed via the test of functional health literacy in adults, and medication knowledge/behavior was assessed via a validated questionnaire (KR-20 = 0.83). Data were analyzed using SPSS (chi-square, *t*-tests, ANOVA, Pearson’s correlation, *P* < 0.05).

**Results:**

It was found that 97.9% of the respondents had adequate health literacy. Age negatively correlated with health literacy (*r* = –0.395; *P* < 0.0001) and positively with medication knowledge (*r* = 0.121; *P* = 0.038). Women exhibited higher health literacy and medication knowledge than men. Higher education levels and living with health professionals were associated with better health literacy. Patients with chronic diseases had lower health literacy than those without (mean scores: 31.5 vs 32.7). Significant correlations were observed between health literacy and treatment adherence and compliance and use.

**Conclusions:**

Targeted health literacy interventions are required across different ages, genders, and education levels to improve medication use and health outcomes. Future research should examine the long-term effects of these interventions.

## 1 Introduction

Health is the most valuable asset in life. Health literacy – an individual’s ability to obtain, comprehend, and use basic health information and services to make informed health decisions – strongly influences health outcomes [1,2,3,4]. Limited health literacy leads to suboptimal healthcare results, including poor adherence to prescribed medical regimens [2,3,4,5]. Inadequate health literacy can lead to difficulty in understanding written medical instructions, particularly those related to medication administration. An individual’s lack of comprehension may lead them to take medications at incorrect dosages, have reduced access to health care, and exhibit noncompliance with prescribed treatments [1,2,6,7].

A higher level of health literacy is associated with better health outcomes and treatment adherence [2,8,9]. Inadequate health literacy often results in suboptimal treatment decision-making, limited understanding of medications, and nonadherence to medical instructions, which can exacerbate health problems [10]. A survey conducted using the short form of the test of functional health literacy in adults (TOFHLA) questionnaire revealed that patients with a low health literacy level and high blood pressure could not identify their blood pressure medications, whereas those with adequate health literacy could identify these medications [8,11,12].

Taiwan’s National Health Insurance data pertaining to the year 2011 indicated that the total healthcare expenditure, including copayments, reached 558.5 billion points. The expenditure for outpatient services reached 384.9 billion points (70%), and it mostly included medical services and material costs, followed by drug expenses. The expenditure for inpatient services was 173.6 billion points (30%), and it predominantly included ward services and subsequent drug expenses. Furthermore, the expenditure for outpatient services at medical centers alone was 81.4 billion points, and it primarily included drug expenses. For outpatient services at regional and local hospitals, the expenditure was 83 and 40 billion points, respectively, and it mainly included medical service and material costs, followed by drug expenses. Regarding inpatient care, the expenditure was primarily for ward services, followed by drug expenses.

Chronic renal failure (uremia), the most common condition in Taiwan, accounts for approximately 47.5% of all outpatient service-related claims. Data from Taiwan’s National Health Insurance Administration of the Ministry of Health and Welfare suggest that Yunlin and Chiayi counties have the highest rates of dialysis worldwide. This trend may be attributable to misleading advertisements that overstate the efficacy of unverified drugs, the public’s susceptibility to such claims, and a high rate of drug misuse. These figures indicate the substantial healthcare burden on the Taiwanese populace. To improve health outcomes and reduce healthcare costs, effective interventions must be developed that improve the public’s understanding and use of medications.

Evidence suggests an association between health literacy and overall health [1,2,3,4]. In consideration of this, the current study investigated how well the residents of southern Taiwan understand and use their medications and how this relates to their health literacy levels.

## 2 Methods

### 2.1 Study design

This study utilized a cross-sectional correlational analysis to examine the relationship between health literacy and individuals’ comprehension and utilization of medications in southern Taiwan. The population using the criteria for inclusion in the study were established as follows: (1) participants must be 20 years of age or older, (2) they must have resided in Chiayi County or City for a minimum duration of 6 months, (3) they should possess the ability to communicate in either Mandarin or Taiwanese, whether verbally or in written form, and (4) they must demonstrate a willingness to participate in the study after receiving a comprehensive explanation of its objectives. The criteria for exclusion included the following: (1) individuals exhibiting impaired consciousness or psychiatric conditions, (2) those with substantial visual or auditory impairments, (3) individuals who opted not to participate, and (4) newly immigrated residents who had resided in Taiwan for less than 6 months. The sample size was determined using G*Power (version 2.1.2), with an effect size of 0.3, an alpha level of 0.05, and a power of 0.8. A total of 102 participants were required to achieve statistical power. To account for potential incomplete responses, 306 individuals were approached, resulting in 300 valid questionnaires (effective response rate: 98%).

### 2.2 Research tools and measurements

This study utilized a structured questionnaire to systematically assess health literacy, medication knowledge, and medication-related behaviors among residents of Chiayi County and Chiayi City. Data collection was conducted from September to November 2013, employing a randomized sampling methodology. Participants were recruited from Chiayi Country and Chiayi City at diverse community events, including temple fairs, local gatherings, public parks, educational institutions, and workplaces. Prior to enrollment, all eligible individuals were thoroughly informed of the study’s objectives, and written consent was obtained to ensure that participation was voluntary and ethically sound.

To evaluate health literacy, the study employed the TOFHLA, a reputable instrument designed to assess both reading comprehension and numerical proficiency. The Taiwanese adaptation of the TOFHLA underwent a comprehensive validation process to establish its reliability and relevance in the target population. A panel of experts meticulously reviewed the questionnaire, resulting in each item receiving a content validity score of 3 points or higher. The content validity index was calculated as 0.85, indicating that 85% of the items were regarded as appropriate for use without requiring any modifications. Concurrent criterion validity was assessed through the utilization of the Chinese version of the Rapid Estimate of Adult Literacy in Medicine [13], which revealed a correlation coefficient of 0.70 (*P* < 0.01). This finding demonstrates a substantial association between the two measurement instruments. The TOFHLA is composed of multiple-choice questions, each with a singular correct answer, and requires approximately 22 min to complete. The scoring range for the TOFHLA extends from 0 to 36, with higher scores indicative of elevated levels of health literacy. Participants were categorized into three distinct levels of health literacy: inadequate (≤16), marginal (17–22), and adequate (≥23).

In addition to assessing health literacy, medication-related knowledge and behaviors were evaluated using the Proper Medication Cognition and Behavior Questionnaire, a newly developed instrument that has undergone rigorous validation. The questionnaire was reviewed by a panel consisting of two senior clinical pharmacists and one senior associate professor, who provided expert feedback for its refinement. The instrument demonstrated high reliability, evidenced by a Kuder–Richardson Formula 20 coefficient (KR-20) of 0.83, indicating strong internal consistency. It evaluates the participants’ understanding of medication indications, proper usage, potential side effects, and adherence to regimens. The questionnaire comprises two subscales: medication cognition (19 items, total score range: 0–19) and medication behavior (15 items, total score range: 0–15). Higher scores indicate a greater accuracy in medication-related knowledge and improved adherence to medication regimens.

Sociodemographic variables were gathered to explore the potential factors that influence health literacy and medication-related behaviors. These variables included age, gender, education level, occupation, the presence of chronic diseases, and whether participants lived with family members employed in healthcare-related professions. Participants were categorized into four age ranges: 20–30, 31–50, 51–64, and 65 years or older. Education levels were classified as illiterate/primary school, high school, and university/graduate school. Occupational data covered various sectors, including agriculture, industry, business, civil service, and service-related professions. Data collection was performed through both face-to-face interviews and self-administered questionnaires, offering flexibility based on the literacy levels and preferences of the participants. Interviewers underwent training to ensure the standardized administration of the survey instruments, thereby reducing the potential for bias in data collection.

### 2.3 Data processing and statistical analysis

All data were meticulously coded and analyzed using SPSS 18.0. Descriptive statistics were employed to summarize the sociodemographic characteristics, health literacy levels, and aspects of medication cognition and behavior. For the inferential statistics, chi-square tests, independent *t*-tests, and one-way ANOVA were utilized to investigate differences across demographic variables. Pearson’s correlation analysis was conducted to explore the relationships between health literacy, medication cognition, and behavior. A *p*-value of less than 0.05 was deemed statistically significant.


**Ethical considerations:** This study was conducted in accordance with the ethical principles of the Declaration of Helsinki. All participants provided informed consent. The study protocol was approved by the Institutional Review Board of Chang Gung Memorial Hospital in Southern Taiwan (permit number 1021637B).

## 3 Results

### 3.1 Sociodemographic characteristics and health literacy levels

The cohort’s demographic and health literacy profiles are presented in Table 1. A total of 300 valid responses were obtained (effective response rate: 98%). Regarding sex distribution, the cohort included 118 men (39.3%) and 182 women (60.6%). Regarding age distribution, 17.0, 50.0, 23.3, and 9.7% of the respondents were aged 20–30, 31–50, 51–64, and 65–80 years, respectively, with significant variations across age categories (*P* < 0.0001). Regarding employment status, 51.3, 22.0, 11.3, 8.3, 4.3, and 2.7% of the respondents were engaged in service industry work, other occupations, business, industrial work, civil service, and agricultural work, respectively, exhibiting significant variations (*P* = 0.0130). Regarding education, 52.3, 32.4, and 13.5% received university or graduate education, high school education, and secondary education or no formal education, respectively (*P* = 0.1854). Furthermore, regarding living with family members having health-related occupations, 69.6% did not reside with such family members, whereas 29% did; approximately 1.3% of the individuals were uncertain of whether they did.

**Table 1:** Sociodemographic attributes and health literacy level of adults in Southern Taiwan

Characteristics	All (%)	Men (%)	Women (%)	*P* value
**Gender**	300 (100)	118 (39.3)	182 (60.6)	—
**Age (years)**				
20–30	51 (17.0)	15 (12.7)	36 (19.8)	<0.0001
31–50	150 (50.00)	48 (40.68)	102 (56.04)	
51–64	70 (23.33)	34 (28.81)	36 (19.78)	
65–80	29 (9.67)	21 (17.80)	8 (4.40)	
**Job occupation**				
Agriculture	8 (2.7)	2 (1.7)	6 (3.3)	0.0130
Industry	25 (8.3)	18 (15.3)	7 (3.9)	
Business	34 (11.3)	15 (12.7)	19 (10.4)	
Civil servants	13 (4.3)	3 (2.5)	10 (5.5)	
Service industry	154 (51.3)	58 (49.2)	96 (52.8)	
Others	66 (22.0)	22 (18.6)	44 (24.2)	
**Education**				
Illiterate–secondary	41 (13.5)	16 (13.6)	25 (13.7)	0.1854
High school	99 (32.4)	46 (39.0)	53 (29.1)	
University–Graduate school	160 (52.3)	56 (47.5)	104 (57.1)	
**Living with family health-related industries**				
Exist	87 (28.4)	33 (28.0)	54 (29.7)	0.4228
Does not exist	209 (68.3)	82 (69.5)	127 (69.8)	
Do not know	4 (1.3)	3 (2.5)	1 (0.6)	
**Health literacy level**				
Insufficient (16↓)	2 (0.7)	1 (0.9)	1 (0.6)	0.0415
Edge (17–22)	4 (1.3)	4 (3.4)	0 (0.0)	
Adequate (23↑)	294 (98.0)	113 (95.8)	181 (99.5)	

Baker et al. used the short-form TOFHLA questionnaire to evaluate health literacy [14]. The respondents’ mean score was 32.24. Inadequate, marginal, and adequate levels of health literacy were noted in 2 (0.7%), 4 (1.3%), and 294 (97.9%) respondents, respectively. Notably, sex significantly influenced the level of health literacy (*P* = 0.0415). The prevalence of chronic diseases was considerably high; the predominant conditions were cardiovascular diseases, such as hypertension, heart disease, and stroke, followed by diabetes and cancer. Similar trends were observed in the participants’ cohabiting family members. Pearson correlation analysis revealed a significant negative correlation between health literacy and age (*r* = −0.395; *P* < 0.0001), indicating that older adults had lower levels of health literacy. Conversely, a positive correlation was noted between age and medication knowledge (*r* = 0.121; *P* < 0.038), suggesting that older adults possessed higher levels of medication knowledge. However, no significant correlation was observed between age and medication use (*r* = 0.024; *P* = 0.678).

### 3.2 Correlation of health literacy with correct medication cognition (CMC) and behavior

A higher level of education was associated with a higher level of health literacy. However, employment exerted no significant effect on health literacy. Living with family members having health-related occupations strongly influenced health literacy. Table 2 presents the correlations of health literacy with correct understanding of and adherence to medications. The level of health literacy was significantly higher in participants adhering to medical instructions than in those not adhering to such instructions (mean score: 32.94 ± 2.45 vs 32.08 ± 3.67; *P* = 0.0034). However, no significant sex-based difference in health literacy was observed between participants adhering to medical instructions and those not adhering to such instructions.

**Table 2:** Analysis of the health literacy correlation between CMC and CMB

Item	All (*N* = 300)			Men (*N* = 118)			Women (*N* = 182)		
	Case (%)	Mean ± SD	*P* value	Case (%)	Mean ± SD	*P* value	Case (%)	Mean ± SD	*P* value
**Health level**									
Obey orders	56 (18.67)	32.94 ± 2.45	0.0034	18 (15.25)	32.06 ± 2.36	0.2219	38 (20.88)	33.37 ± 2.41	0.2086
Does not obey orders	244 (81.33)	32.08 ± 3.67		100 (84.75)	31.18 ± 4.38		144 (79.12)	32.71 ± 2.98	
**CMC**									
Obey orders	56 (18.67)	17.66 ± 1.18	0.7645	18 (15.25)	17.72 ± 1.45	0.2597	38 (20.88)	17.63 ± 1.05	0.1637
Does not obey orders	244 (81.33)	17.60 ± 1.77		100 (84.75)	17.14 ± 2.09		144 (79.12)	17.92 ± 1.42	

A *t-*test revealed significant effects of sex on health literacy (*P* < 0.0001); the level of health literacy was higher in women than in men. Moreover, chronic diseases significantly influenced health literacy (*P* < 0.0001). However, no significant difference in health literacy was observed between respondents whose family members had chronic diseases and those whose family members did not have such diseases (*P* = 0.155). A significant sex-based difference was noted in medication knowledge (*P* < 0.003), with women outperforming men. Furthermore, a significant difference in the understanding of medication was noted between individuals with chronic diseases and those without such diseases (*P* < 0.0001). No significant difference was observed between respondents whose family members had chronic diseases and those whose family members did not (*P* = 0.317). Univariate analysis revealed a significant positive correlation between health literacy and proper medication knowledge (*r* = 0.520; *P* < 0.0001), indicating that a higher level of health literacy was associated with a higher level of medication knowledge. However, no significant correlation was observed between health literacy and proper medication use (*r* = −0.014; *P* = 0.804). Furthermore, no significant correlation was noted between medication knowledge and medication use (*r* = 0.093; *P* = 0.109).

### 3.3 Correct health behaviors, medication cognition, and health literacy

The average health literacy score was significantly higher in participants adhering to medical instructions than in those not adhering to instructions (32.9 vs 32.08; *P* ≤ 0.05). However, no significant difference in medication knowledge was observed between compliant and noncompliant participants (17.66 vs 17.60; *P* = 0.123). Table 3 presents the correct health behaviors, medication cognition, and health literacy levels in the patients stratified by demographic characteristics. Health literacy was significantly influenced by age (*F* = 21.873; *P* < 0.01) and education level (*F* = 27.963; *P* < 0.01). The level of health literacy was the highest in participants with a university or graduate education level (mean score: 33.23) and the lowest in those with a secondary education level or no formal education (mean score: 29.07).

**Table 3:** Correct health behaviors, CMC, and health literacy for residents in Southern Taiwan

Characteristics	Health literacy level		CMC		CMB	
	Average score	*F* value	Average score	*F* value	Average score	*F* value
**Age (years)**	32.24	21.873(**)	17.61	4.548(*)	11.98	1.781
20–30	33.04		17.82		11.86	
31–50	33.21		17.85		11.77	
51–64	31.14		17.30		12.29	
65–80	28.52		16.79		12.55	
**Job occupation**	32.24	1.483	17.61	0.748	11.98	0.603
Agriculture	31.26		17.39		12.24	
Industry	33.43		18.00		12.43	
Business	32.28		17.28		11.88	
Civil servants	32.56		17.79		12.21	
Service industry	32.69		18.00		11.46	
Others	32.49		17.67		11.86	
**Education**	32.23	27.963(**)	17.61	12.989(**)	11.98	3.033(*)
Illiterate–secondary	29.07		16.56		12.37	
High school	31.94		17.47		12.26	
University–graduate school	33.23		17.97		11.70	
**Living with family health-related industries**	32.24	5.841(**)	17.61	1.254	11.98	1.223
Exist	32.25		17.55		12.05	
Does not exist	32.49		17.80		11.87	
Do not know	26.50		16.75		10.50	

*P < 0.05, **P < 0.01.

### 3.4 Correlation between CMC and correct medication behavior (CMB)

Individuals’ medication purchase behaviors are influenced by recommendations from family members and friends and information from sources such as television commercials, radio advertisements, the Internet, public parks, and traditional markets. In the current study, a significant positive correlation was observed between proper medication knowledge and correct medication use (*r* = 0.378; *P* < 0.0001), indicating that a higher level of medication knowledge was associated with better use of medications. A positive correlation was noted between CMC and CMB (*r* = 0.09; Table 4). This correlation was slightly stronger in women than in men (*r* = 0.13 vs 0.09).

**Table 4:** Analysis of the correlation between CMC and CMB

Item	All (*N* = 300)		Men (*N* = 118)		Women (*N* = 182)	
	CMC	CMB	CMC	CMB	CMC	CMB
CMC	1	0.09	1	0.09	1	0.13
CMB	0.09	1	0.09	1	0.13	1

CMC: correct medication cognition.

CMB: correct medication behavior.

## 4 Discussion

We investigated the associations of health literacy with individuals’ understanding and use of medications. Our findings revealed a negative association between age and health literacy (*r* = −0.395; *P* < 0.0001) and a positive correlation between age and medication knowledge (*r* = 0.121; *P* = 0.038). However, no significant association was noted between age and medication behavior (*r* = 0.024; *P* = 0.678). Furthermore, health literacy was significantly and positively correlated with CMC (*r* = 0.520; *P* < 0.0001) but not medication behavior (*r* = −0.014; *P* = 0.804).

Our findings are consistent with those of other studies indicating significant effects of health literacy on health behaviors and outcomes [5,15,16]. Evidence suggests that individuals with low health literacy levels often struggle to comprehend medical instructions, leading to improper medication use and poor health outcomes [3,7,17]. The present study adds to the current literature by unveiling the intricate associations between health literacy, medication knowledge, and medication behavior across age groups. These findings underscore the urgent need for tailored interventions to address health literacy issues.

Although we identified a negative correlation between age and health literacy, a positive correlation was noted between age and medication knowledge. Older adults may acquire medication knowledge through interactions with healthcare systems and providers over time. The low level of health literacy in the older population may be attributable to generational disparities in education and access to health information [18,19]. This disparity suggests that although older adults possess medication knowledge, they lack the broader health literacy required for effective health management. This finding highlights the need for age-specific health literacy interventions.

Our findings revealed sex disparities, indicating that women outperform men in terms of health literacy and medication knowledge. This may be attributable to traditional gender roles, with women often shouldering the responsibility of overseeing family health. Thus, women proactively seek and comprehend health information, which ultimately increases their health literacy and medication knowledge [20,21]. Acknowledging sex-based differences is essential in developing health literacy interventions tailored to the unique requirements and strengths of each sex. This underscores the importance of addressing gender disparities in health literacy through sex-specific interventions.

Our findings highlight the significant role of family influence in health literacy. Living with family members having health-related occupations increased the level of health literacy, indicating that exposure to health information and practices within the family unit can markedly improve an individual’s health literacy [22,23]. This suggests a potential for positive change through family-based health literacy interventions, which could leverage the influence of health-literate family members and create a supportive learning environment.

Education level significantly influenced health literacy: a higher level of education was correlated with a higher level of health literacy. This correlation aligns with the premise that education nurtures critical thinking and information-processing skills, which are pivotal for understanding and using health information [9,24,25,26,27,28]. Thus, educational interventions may help improve health literacy, particularly in individuals with low educational achievements. Educational interventions aimed at enhancing health literacy should be incorporated into school curricula and adult education programs. These interventions should focus on cultivating the skills required to navigate the healthcare system and to understand health information. Furthermore, health-literate family members should be involved in these interventions to create a supportive environment for acquiring and applying health-related knowledge.

## 5 Limitations

This study has some limitations. The applicability of our findings, which were derived from a sample from southern Taiwan to other regions or populations may be low. Future studies should validate our findings in different contexts by including a diverse sample. Furthermore, the cross-sectional design of this study precluded the establishment of causal relationships. Thus, longitudinal studies are required to clarify the dynamic interplay between health literacy, medication knowledge, and medication behavior over time. Future studies should measure the effects of targeted health literacy interventions on medication behavior and health outcomes; findings from such studies may provide substantial evidence for effective practices. Furthermore, in the current era of telehealth and online health information, researchers should investigate the benefits of digital health literacy to guide the development of novel strategies for enhancing health literacy.

## 6 Conclusions

This study highlights the importance of health literacy in understanding and adhering to medication regimens. It further highlights the need for targeted health education and interventions to cater to the unique requirements of various demographic groups, thereby achieving the goal of enhancing health literacy and thus improving health outcomes for all.

## 7 Implications

This study has several practical implications. First, interventions to enhance health literacy should be tailored to the specific needs of older adults, who may require extensive support to bridge the gap between medication knowledge and health literacy. Second, sex-specific interventions should be developed to accommodate men's and women’s distinct approaches to processing health information. Finally, healthcare practitioners should recognize their patients’ diverse levels of health literacy and adopt strategies to ensure the accessibility and comprehensibility of health information for all, irrespective of age, sex, or education level. Such strategies may involve the use of plain language and visual aids and reinforcement of comprehension through teach-back techniques.
